# Impact of Physician Specialty on Quality Care for Patients Hospitalized with Decompensated Cirrhosis

**DOI:** 10.1371/journal.pone.0123490

**Published:** 2015-04-02

**Authors:** Nicholas Lim, Steven D. Lidofsky

**Affiliations:** 1 Division of Gastroenterology and Hepatology, University of Vermont College of Medicine, Burlington, Vermont, United States of America; 2 University of Vermont Medical Center, Burlington, Vermont, United States of America; University of Sydney, AUSTRALIA

## Abstract

**Background:**

Decompensated cirrhosis is a common precipitant for hospitalization, and there is limited information concerning factors that influence the delivery of quality care in cirrhotic inpatients. We sought to determine the relation between physician specialty and inpatient quality care for decompensated cirrhosis.

**Design:**

We reviewed 247 hospital admissions for decompensated cirrhosis, managed by hospitalists or intensivists, between 2009 and 2013. The primary outcome was quality care delivery, defined as adherence to all evidence-based specialty society practice guidelines pertaining to each specific complication of cirrhosis. Secondary outcomes included new complications, length-of-stay, and in-hospital death.

**Results:**

Overall, 147 admissions (59.5%) received quality care. Quality care was given more commonly by intensivists, compared with hospitalists (71.7% vs. 53.1%, P = .006), and specifically for gastrointestinal bleeding (72% vs. 45.8%, P = .03) and hepatic encephalopathy (100% vs. 63%, P = .005). Involvement of gastroenterology consultation was also more common in admissions in which quality care was administered (68.7% vs. 54.0%, P = .023). Timely diagnostic paracentesis was associated with reduced new complications in admissions for refractory ascites (9.5% vs. 46.6%, P = .02), and reduced length-of-stay in admissions for spontaneous bacterial peritonitis (5 days vs. 13 days, P = .02).

**Conclusions:**

Adherence to quality indicators for decompensated cirrhosis is suboptimal among hospitalized patients. Although quality care adherence appears to be higher among cirrhotic patients managed by intensivists than by hospitalists, opportunities for improvement exist in both groups. Rational and cost-effective strategies should be sought to achieve this end.

## Introduction

Health care reform has placed a premium on quality-based practice [1], especially among hospitalized patients [2]. One of the most common conditions that leads to hospitalization is decompensated cirrhosis, manifestations of which include gastrointestinal (GI) variceal bleeding, ascites, and encephalopathy [3]. Although the prevalence of cirrhosis is rising in response to an aging population with chronic liver disease [4,5], comparatively little is known about quality care in cirrhosis, in contrast to other disorders that trigger hospitalization [6].

Three recent studies have examined adherence to cirrhosis-related quality indicators, which were derived from society-based practice guidelines [7,8], and independent panels of experts [9]. Two focused on management of ascites and of gastroesophageal varices in Veterans Administration (VA) ambulatory and hospital settings [10,11], and one examined the proportion of quality indicators met for specific complications of cirrhosis in patients hospitalized at a single center [12]. A striking finding was that a high percentage of cirrhotic patients did not receive quality care for disease complications. In the single center study, the proportion of quality indicators met in hospitalized patients averaged 45% [12], and in the VA studies, quality indicator adherence was as low as 30% [10,11]. The VA studies also showed that involvement of a gastroenterology specialist was associated with increased adherence to quality indicators for ascites and for varices, although this was not analyzed specifically for inpatients [10,11]. It remains unaddressed in patients hospitalized for complications of cirrhosis whether gastroenterology consultation or the specialty of the managing physician influences quality care.

In many medical centers, patients are managed by hospitalists or critical care specialists (intensivists) [13,14]. Although hospitalists and intensivists achieve similar outcomes in comparable clinical settings, in selected critical conditions, superior outcomes are conferred by intensivists [15]. We therefore speculated that differences between intensivists and hospitalists may also be present with respect to quality care delivery to hospitalized cirrhotic patients and tested whether adherence to guidelines for decompensated cirrhosis is higher in patients who are admitted by intensivists, compared with hospitalists.

## Methods

### Study population

We reviewed the electronic medical records of patients admitted to the University of Vermont Medical Center. Here, patients with decompensated cirrhosis are admitted to the medical intensive care unit (managed exclusively by intensivists), or to general medical units (managed exclusively by hospitalists). Subjects were identified using hospital discharge diagnosis codes (571.2 Alcohol Cirrhosis Liver; 571.5 Cirrhosis of Liver NOS; and 571.6 Biliary Cirrhosis). The study design was approved by the University of Vermont Committee on Human Research in the Medical Sciences (CHRMS M13-216). A waiver of informed consent was obtained, since the data were analyzed from the electronic medical record and are reported without personal identifiers.

Inclusion criteria were: (a) admission to hospital with a complication of cirrhosis (refractory ascites, gastrointestinal bleeding, hepatic encephalopathy, or spontaneous bacterial peritonitis) and (b) evidence of cirrhosis established by liver biopsy, radiological detection of a nodular liver, or biochemical abnormalities (e.g., hyperbilirubinemia, prolonged prothrombin time). Exclusion criteria were: (a) age less than 18 years, (b) transfer from an outside institution more than 24 h after initial presentation, (c) admission for reasons other than a complication of cirrhosis, (d) concurrent hepatocellular carcinoma, or (e) advanced directives that may have reduced the likelihood of aggressive care measures. The severity of liver disease at the time of admission was assessed by the Model for End-stage Liver Disease (MELD) score [16].

### Outcomes

The primary outcome was adherence to quality care, which we defined as adherence to all quality indicators that were derived from the highest-level society-based clinical practice guidelines (Table 1) relevant to the condition responsible for admission [7,8,17,18]. Secondary outcomes included in-hospital complications (defined as a new problem distinct from the admitting diagnosis, S1 Table), transfer (when applicable) from a general medical unit to the intensive care unit (ICU), length of hospital stay, and in-hospital death.

**Table 1:** Evidence-based quality measures for specific complication of cirrhosis.

Refractory Ascites [8,18]
• Diagnostic paracentesis in a timely manner
• Aspirated fluid sent for cell count, differential and culture
• Management with diuretics and sodium restriction in patients with normal renal function
Upper GI Bleeding [7]
• Endoscopy (EGD) within 12 hours
• Administration of antibiotics prior to EGD
• In cases of variceal hemorrhage, use of somatostatin analogues
• In cases of variceal hemorrhage, use of endoscopic band ligation or sclerotherapy
• In cases of re-bleeding, repeat EGD or transjugular intrahepatic portosystemic shunt
Hepatic Encephalopathy [17]
• Evidence of search for reversible factors of hepatic encephalopathy
• Diagnostic paracentesis if ascites documented
• Treatment with lactulose or rifaximin in cases of persistent hepatic encephalopathy
Spontaneous Bacterial Peritonitis [8,18]
• Diagnostic paracentesis in a timely manner
• Administration of empiric antibiotics within 6 hours following diagnosis of spontaneous bacterial peritonitis
• Administration of intravenous albumin within 6 hours on day 1 and on day 3 following diagnosis of spontaneous bacterial peritonitis, in patients with serum creatinine > 1.0 mg/dL, BUN > 30 mg/dL, or bilirubin > 4.0 mg/dL

### Statistics

Analyses were performed with GraphPad Prism (version 6.0). Differences between groups were determined by Fisher’s exact test for categorical variables and by Mann-Whitney test for numerical variables. A value of P <. 05 was deemed statistically significant.

## Results

### Characteristics of the study population

Between June 2009 and July 2013, there were 1375 consecutive admissions with cirrhosis. Of these, 247 met the study entry criteria. Reasons for exclusion included admission for reasons other than decompensated cirrhosis (383 admissions); arrival from an outside institution more than 24 hours after presenting with symptoms of decompensated liver disease (159 admissions) and miscellaneous conditions as described above (586 admissions).

As shown in Table 2, the median age of the study population was 56 years; 143 (57.9%) of admissions were in men, the median admission MELD score was 17, and the most common diagnosis was alcoholic cirrhosis (51.8%). The principal reasons for admission were refractory ascites (n = 39, mean admission MELD score = 17.34), upper GI bleeding (n = 92, mean MELD score = 14.54), hepatic encephalopathy (n = 83, mean admission MELD score = 18.18), and spontaneous bacterial peritonitis (n = 33, mean admission MELD score = 21.31).

**Table 2:** Baseline characteristics of the study population.

	Overall (247)	Hospitalist (162)	Intensivist (85)	P-value
Median age (range), years	56 (26–84)	57 (26–84)	54 (26–78)	0.04
Male (%)	143 (57.9)	90 (55.5)	53 (62.3)	0.34
Median MELD score (range)	17 (6–47)	17 (7–37)	15 (6–47)	.02
Etiology of cirrhosis (%)				
Alcohol	128 (51.8)	79 (48.8)	49 (57.7)	0.23
Alcohol & hepatitis C	49 (19.8)	34 (21)	15 (17.7)	0.62
Hepatitis C	20 (8.1)	13 (8)	7 (8.2)	>.99
NAFLD	17 (6.9)	10 (6.2)	7 (8.2)	0.6
Other	33 (13.4)	26 (16)	7 (8.2)	0.11
Reason for admission (%)				
Refractory Ascites	39 (15.8)	36 (22.2)	3 (3.5)	0.0001
Upper GI Bleeding	92 (37.2)	24 (14.8)	68 (80)	0.0001
Hepatic Encephalopathy	83 (33.6)	73 (45.1)	10 (11.8)	0.0001
Spontaneous Bacterial Peritonitis	33 (13.4)	29 (17.9)	4 (4.7)	0.003

NOTE: Data are presented for the entire study population (overall admissions), hospitalist-managed admissions, and intensivist-managed admissions. P-value refers to comparisons between hospitalist versus intensivist-managed admissions. For etiology of cirrhosis, NAFLD: nonalcoholic fatty liver disease, and other includes (but is not limited to) autoimmune hepatitis, primary sclerosing cholangitis, α-1 anti-trypsin deficiency and cardiac cirrhosis.

The majority (65.6%) of admissions were managed initially by hospitalists. There was a small statistically significant increase in the median age and MELD scores of these admissions (compared with intensivist-managed admissions), and admissions for GI bleeding were more commonly managed by intensivists (Table 2). By contrast, hospitalists more commonly initially managed admissions for other complications of cirrhosis. Gastroenterology consultation was obtained in 62.8% of admissions, and all admissions with GI bleeding received gastroenterology consultation. Gastroenterology consultation was obtained in a significantly higher proportion of intensivist-managed admissions, compared with those managed by hospitalists (90.5% vs. 48%, P <. 001).

### Adherence to quality care and relation to physician specialty

Quality care criteria were met in 147 of the 247 admissions for decompensated cirrhosis (59.5%), and the baseline characteristics of these admissions (and those in which such criteria were not met) are presented in S2 Table. Overall, a significantly higher proportion of intensivist-managed admissions, compared with those managed by hospitalists, met criteria for adherence to quality care indicators (71.7% vs. 53.1%, P = .006), specifically for upper GI bleeding, (72% vs. 45.8%, P = .03), and for hepatic encephalopathy (100% vs. 63%, P = .03), but not for refractory ascites or spontaneous bacterial peritonitis (Table 3).

**Table 3:** Proportion of patients receiving quality care based on admitting physician specialty.

	Hospitalist (%)	Intensivist (%)	P-value
Refractory Ascites	19/36 (52.7)	1/3 (33.3)	0.61
Upper GI Bleeding	11/24 (45.8)	49/68 (72)	0.03
Hepatic Encephalopathy	46/73 (63)	10/10 (100)	0.03
Spontaneous Bacterial Peritonitis	10/29 (34.4)	1/4 (25)	>0.99
Overall	86/162 (53.1)	61/85 (71.7)	.006

NOTE: Hospitalist denotes admissions by hospitalists satisfying study criteria for quality care for a particular complication of decompensated cirrhosis. Intensivist denotes admissions by intensivists satisfying study criteria for quality care for a particular complication of decompensated cirrhosis. Hospitalists and intensivists each had access to bedside ultrasound and medications for UGI bleeding, including octreotide.

Gastroenterology consultation was obtained in a significantly higher proportion of admissions that met quality care criteria (68.7% vs. 54.0%, P = .023). Among intensivist-managed admissions, adherence to quality indicators did not significantly differ in the presence versus absence of gastroenterology consultation (S3 Table). By contrast, among hospitalist-managed admissions, gastroenterology consultation was associated with a significantly higher adherence to quality indicators for hepatic encephalopathy (86.9% vs. 52%, p = .004), but not for other complications of cirrhosis (S4 Table).

### Adherence to quality care and clinical outcomes

Death occurred in 11 of the 247 admissions (4.5%). New complications developed in 48 admissions (19.4%), and the median length of stay was 4 days (range 1–63). There was a non-significant trend toward reduced inpatient mortality among admissions that met criteria for quality care, compared with those that did not (2% vs. 8%, P = .054), and no significant differences were detected with respect to the development of new complications or length of hospitalization (Table 4). To examine these findings in more detail, we analyzed the relation between adherence to quality indicators and the clinical outcomes of admissions for refractory ascites, upper GI bleeding, hepatic encephalopathy, and spontaneous bacterial peritonitis.

**Table 4:** Adverse clinical outcomes and relation to adherence to quality care.

	Quality care (%)	Non-quality care (%)	P-value
**Overall**			
In-hospital complications	25/147 (17)	23/100 (23)	0.25
ICU transfer, where eligible	12/88 (13.6)	7/74 (9.4)	0.47
Length of hospital stay, median	4	4	0.76
In-hospital deaths	3/147 (2)	8/100 (8)	0.054
**Refractory Ascites**			
In-hospital complications	2/20 (10)	7/19 (36.8)	0.06
ICU transfer, where eligible	2/19 (10.5)	2/17 (11.8)	>0.99
Length of hospital stay, median	3	3	0.74
In-hospital deaths	1/20 (5)	4/19 (21)	0.18
**Upper GI Bleeding**			
In-hospital complications	13/60 (21.6)	6/32 (18.8)	0.79
ICU transfer, where eligible	5/12 (41.6)	1/12 (8.3)	0.16
Length of hospital stay, median	4	3	0.40
In-hospital deaths	1/60 (1.66)	1/32 (3.12)	>0.99
**Hepatic Encephalopathy**			
In-hospital complications	8/56 (14.3)	5/27 (18.5)	0.75
ICU transfer, where eligible	5/47 (10.6)	3/27 (11.1)	>0.99
	Quality care (%)	Non-quality care (%)	P-value
Length of hospital stay, median	4	4	0.90
In-hospital deaths	1/56 (1.78)	0/27 (0)	>0.99
**Spontaneous Bacterial Peritonitis**			
In-hospital complications	2/11 (18)	6/22 (23)	>0.99
ICU transfer, where eligible	0/8 (0)	1/20 (5)	>0.99
Length of hospital stay, median	5	6.5	0.44
In-hospital deaths	0/11 (0)	3/22 (13.6)	0.53

NOTE: Quality care denotes admissions satisfying study definition of quality care. Non-quality care denotes admissions not satisfying study definition of quality care.

#### Refractory ascites

Among the 39 admissions for refractory ascites, the overall inpatient mortality was 12.8%, and acute kidney injury was the most common in-hospital complication (5 out of 9). Twenty of these admissions met quality care criteria (51.3%). Adherence to quality indicators for ascites was associated with a non-significant trend toward reduced in-hospital complications that were not present on admission (10% vs. 36.8%, P = .06). Significant differences were not detected, however, between admissions that met quality care criteria and those that did not, with respect to other clinical outcomes (Table 4).

Sub-group analysis was performed to determine the influence on clinical outcomes of adherence to specific quality care measures for ascites. Of the 19 admissions that did not meet quality criteria, paracentesis was not performed in a timely fashion in 15 (78.9%). This was the only quality measure for ascites that was associated with significant differences in clinical outcomes (S5 Table). Among admissions in which diagnostic paracentesis was performed promptly, new complications were significantly reduced, compared with those in which paracentesis was delayed (9.5% vs. 46.6%, P = .02).

### Upper GI bleeding

Inpatient mortality among the 92 admissions for upper GI bleeding was 2%, and 65.2% of these admissions for met criteria for quality care (Table 4). Neither mortality nor development of new complications significantly differed between admissions in which quality care criteria were met and those that did not (Table 4). Administration of antibiotics prior to endoscopy was the only specific quality measure for upper GI bleeding that was associated with statistically significant differences in clinical outcomes (S5 Table); median length of hospitalization (compared with no pre-procedural antibiotics) was increased (4 days vs. 3 days, P = .04).

### Hepatic encephalopathy

The in-hospital mortality of the 83 admissions for hepatic encephalopathy was 1.2%; 67.5% met quality care criteria. Among the 27 admissions in which such criteria were not met, evaluation for reversible factors of hepatic encephalopathy was omitted in 26 (96%). However, neither adherence to individual nor to all quality indicators significantly affected clinical outcomes (S5 Table).

### Spontaneous bacterial peritonitis

Of the 33 admissions for spontaneous bacterial peritonitis, mortality was 9%, in-hospital complications occurred in 21.2%, and 33.3% met quality care criteria. Acute kidney injury complicated 6 admissions, and hepatic encephalopathy complicated 3 admissions. Statistically significant differences in clinical outcomes could not be detected between admissions that met all quality care criteria for spontaneous bacterial peritonitis, compared with those that did not (Table 4). However, omission of a timely diagnostic paracentesis was associated with a significantly increased median length of hospital stay (5 days versus 13 days, P = .02, see S5 Table).

## Discussion

Despite the availability of established practice guidelines for cirrhosis [7,8,17,18], a substantial proportion of hospitalized cirrhotic patients miss opportunities for quality care interventions for disease-specific complications. Our findings, in which fewer than 60% of admissions met quality care criteria, are consistent with those of others [10–12]. Here we have provided new insight concerning how admitting physician specialty might influence quality care in this patient population. Among admissions to our center for decompensated cirrhosis, adherence to practice guideline-based quality indicators was higher: (a) when managed by intensivists than hospitalists, specifically for upper GI bleeding and hepatic encephalopathy, and (b) with involvement of gastroenterology consultants.

Why might have practice guideline adherence differed between intensivists and hospitalists? One potential explanation is that there are differences between intensivists and hospitalists in the length and content of postgraduate education [19–21]. In particular, in order to be eligible for specialty certification by the American Board of Internal Medicine, intensivists must pursue at least two more years of mentored clinical training, at least one of which is devoted to critical care medicine, beyond the typical duration of formal training for hospitalists. This could foster increased awareness, by intensivists in training, of practice guidelines as they relate to disorders, such as cirrhosis, which challenge critical care medicine. Another possibility is that the location (rather than admitting physician specialty) influences utilization of quality care measures. The ICU setting might be expected to facilitate interventions, such as prompt administration of medications and on-site procedures, for cirrhotic patients. This could translate into more common recruitment of gastroenterology consultants by intensivists, as we have observed. However, because nearly all intensivist-managed admissions received gastroenterology consultation, it was not possible to demonstrate that involvement of a gastroenterology consultant was independently associated with increased adherence to quality indicators for decompensated cirrhosis in the ICU, as we observed for hospitalist-managed admissions for hepatic encephalopathy. Support for a positive contribution of gastroenterology consultation to quality care in hospitalized cirrhotic patients comes from a study that demonstrated that gastroenterology consultation was associated with significantly reduced inpatient mortality, length of stay and readmission rate at an urban VA hospital [22]. Building upon this idea, a large tertiary medical center has tested joint management by hospitalists and hepatologists for cirrhotic inpatients, which increased quality care for spontaneous bacterial peritonitis [23].

Although adherence to evidence-based quality indicators is expected to improve clinical outcomes, this has been challenging to confirm among cirrhotic patients. In one study, the proportion of quality indicators implemented for specific cirrhosis-related complications did not appear to influence mortality or length of hospitalization [12]. In our study, adherence to quality measures was associated with a non-significant trend toward reduction in hospital mortality, and like the prior study, did not appear to influence length of stay. On closer inspection, adherence to two specific quality measures, diagnostic paracentesis in ascites-related admissions, and antibiotic administration in upper GI bleeding, was associated with significant differences in selected clinical outcomes.

Timely diagnostic paracentesis was associated with significant reductions in new complications among admissions for refractory ascites and in hospitalization duration among admissions for spontaneous bacterial peritonitis. Although we were not able to show that timely paracentesis influenced mortality among these admissions, this may have been limited by our sample size. Indeed, analysis of a multihospital discharge database suggests that timely paracentesis reduces inpatient mortality in cirrhotics with ascites [24]. An unexpected finding in our study was that antibiotic use in upper GI bleeding was associated with an increased length of hospital stay. It is possible that antibiotic usage triggered complications (e.g., diarrhea, rash) that prolonged hospitalization. Alternatively, sicker patients, destined to have longer hospital stays, may have received antibiotics preferentially.

Several additional points merit comment. First, it should be emphasized that the inpatient mortality in our cohort was low, especially with upper GI bleeding: 2% in our series, compared with the published 15% mortality rate for gastroesophageal variceal hemorrhage [25]. A potential explanation is that we did not analyze outcomes of admissions in which transfer from outside institutions (and potential access to higher quality care) was delayed. Second, in contrast with prior studies [10–12], we used an all-or-none definition of quality care, with respect to adherence to guidelines for specific complications of cirrhosis. Third, we confined our selection of quality indicators to those relevant to inpatient care (as opposed to future outpatient management) and where possible, to those with a high level of supportive evidence. Although practice guidelines for ascites and spontaneous bacterial peritonitis were revised during the study time period [8,18], the quality indicators we selected did not change. The one exception to our selection of evidence-based quality indicators was for hepatic encephalopathy, for which the American College of Gastroenterology guidelines (available during the time period of the study) did not use a formal grading system [17]. These indicators have now been examined by the AASLD and European Association for the Study of the Liver, and the corresponding guidelines have been graded as high quality and strongly recommended [26]. Fourth, because our study was retrospective, we could examine associations between physician specialty and quality care but not make inferences about causality, and the sample size limits the statistical power to examine the impact of quality care on all clinical outcomes. These caveats aside, our findings suggest that there are opportunities for quality improvement, regardless of physician specialty, in the care of the hospitalized patient with cirrhosis.

An area that is ripe for exploration is to examine the clinical impact of tools that increase quality indicators adherence in hospitalized cirrhotic patients. Precedent for this exists in the use of standardized order sets, which have been shown to improve clinical outcomes in sepsis [27], and in a pilot nonrandomized study [28], to demonstrate improvements in the use and time to administration of antibiotics in cirrhotic patients hospitalized with upper GI bleeding. Extension of this concept to hospitalizations for other complications of cirrhosis could improve quality care and is worthy of investigation.

## Supporting Information

S1 Table Definitions of in-hospital complications. (DOCX)

S2 Table Baseline characteristics based on receipt of quality care. (DOCX)

S3 Table Provision of quality care in intensivist-managed admissions that did or did not receive gastroenterology (GI) consultation. (DOCX)

S4 Table Provision of quality care in hospitalist-managed admissions that did or did not receive gastroenterology (GI) consultation. (DOCX)

S5 Table Proportion of adverse clinical outcomes in relation to adherence to specific quality measures. (DOCX)
