# Supplementary material for: Impact of Physician Specialty on Quality Care for Patients Hospitalized with Decompensated Cirrhosis
Source: PLoS One. 2015 Apr 2;10(4):e0123490. doi: 10.1371/journal.pone.0123490 (PMC4383455; doi:10.1371/journal.pone.0123490)
Supplement: S1 Table — (DOCX) [file pone.0123490.s001.docx]

Table S1. Definitions of in-hospital complications

| 1. Gastrointestinal bleeding (fresh hematemesis or nasogastric aspiration of ≥100 ml of fresh blood ≥2 h after the start of a specific drug treatment or therapeutic endoscopy; development of hypovolemic shock; 3g drop in hemoglobin within any 24 h period if no transfusion is administered)^1^ |
| --- |
| 1. Spontaneous bacterial peritonitis (an elevated peritoneal fluid neutrophil count (i.e., 250 cells/mm3) without an evident intra-abdominal, surgically treatable source of infection)^2^ |
| 1. Hepatic encephalopathy (a reversible deterioration in neurological function in the absence of an acute neuropsychiatric event)^3^ |
| 1. Acute kidney injury (an increase in serum creatinine of 0.3 mg/dL or a 50% rise from baseline)^4^ |
| 1. Sepsis (defined as systemic inflammatory response syndrome (SIRS)- two of 1) body temperature greater than 38°C, or less than 36°C; 2) a heart rate greater than 90 beats per minute; 3) tachypnea, manifested by respiratory rate greater than 20 breaths per minute, or hyperventilation, as indicated by a PaCO_2_ of less than 32 mmHg or 4) an alteration in the white blood cell count, such as a count greater than 12,000/mm^3^, a count less than 4,000/mm^3^, or the presence of more than 10 percent immature neutrophils (“bands”)- as a result of a confirmed infectious process)^5^ |
| 1. Falls (an event reported either by the faller or a witness, resulting in a person inadvertently coming to rest on the ground or another lower level, with or without loss of consciousness or injury)^6^ |
| 1. Mechanical ventilation (the need for artificial oxygenation and ventilation due to inability to protect one’s airway and/or inability to maintain adequate oxygen saturation) |
| 1. Aspiration pneumonia/pneumonitis (pulmonary infiltrate in the presence of a) at least on respiratory symptom (cough, dyspnea, sputum production or pleuritic pain with b) at least one finding on auscultation (rales or crepitations) or one sign of infection (core body temperature >38°C or <36°C, shivering or leukocyte count >10000/mm^3^ or <4000/mm^3^ related to an aspiration event or suspected aspiration event)^7^ |

1. de Franchis R. Revising consensus in portal hypertension: report of the Baveno V consensus workshop on methodology of diagnosis and therapy in portal hypertension. J Hepatol. 2010;53: 762-768.

2. Runyon BA. Management of adult patients with ascites due to cirrhosis: an update. Hepatology. 2009;49: 2087-2107.

3. Blei AT, Cordoba J. Hepatic Encephalopathy. Am J Gastroenterol. 2001;96: 1968-1976.

4. Belcher JM, Garcia-Tsao G, Sanyal AJ, Bhogal H, Lim JK, Ansari N, et al. Association of AKI with mortality and complications in hospitalized patients with cirrhosis. Hepatology. 2013;57: 753-762.

5. Bone RC, Balk RA, Cerra FB, Dellinger RP, Fein AM, Knaus WA, et al. Definitions for sepsis and organ failure and guidelines for the use of innovative therapies in sepsis. The ACCP/SCCM Consensus Conference Committee. American College of Chest Physicians/Society of Critical Care Medicine. 1992. Chest. 2009;136: e28.

6. Sanders KM, Stuart AL, Williamson EJ, Simpson JA, Kotowicz MA, Young D, et al. Annual high-dose oral vitamin D and falls and fractures in older women: a randomized controlled trial. JAMA. 2010;303: 1815-1822.

7. Bajaj JS, O'Leary JG, Reddy KR, Wong F, Olson JC, Subramanian RM, et al. Second infections independently increase mortality in hospitalized patients with cirrhosis: the North American consortium for the study of end-stage liver disease (NACSELD) experience. Hepatology. 2012;56: 2328-2335.
