# Supplementary material for: Impact of Physician Specialty on Quality Care for Patients Hospitalized with Decompensated Cirrhosis
Source: PLoS One. 2015 Apr 2;10(4):e0123490. doi: 10.1371/journal.pone.0123490 (PMC4383455; doi:10.1371/journal.pone.0123490)
Supplement: S2 Table — (DOCX) [file pone.0123490.s002.docx]

Table S2. Baseline characteristics based on receipt of quality care

|  | Quality care (147) | Non-quality care (100) | P-value |
| --- | --- | --- | --- |
| Median age (range), years | 56 (36-82) | 55.5 (26-84) | 0.87 |
| Male (%) | 84 (57.1) | 59 (59) | 0.79 |
| MELD on admission, median (range) | 16 (6-47) | 18 (7-34) | 0.18 |
|  |  |  |  |
| Etiology of cirrhosis (%) |  |  |  |
| Alcohol | 72 (48) | 56 (56) | 0.30 |
| Alcohol & hepatitis C | 29 (20) | 20 (19.6) | >0.99 |
| Hepatitis C | 12 (8) | 8 (8) | >0.99 |
| NAFLD | 13 (8.8) | 4 (4) | 0.20 |
| Other | 21 (14.3) | 12 (12) | 0.70 |
|  |  |  |  |
| Reason for admission (%) |  |  |  |
| Refractory Ascites | 20 (14) | 19 (19) | 0.29 |
| Upper GI Bleeding | 60 (41) | 32 (32) | 0.18 |
| Hepatic Encephalopathy | 56 (38) | 27 (27) | 0.08 |
| Spontaneous Bacterial Peritonitis | 11 (7) | 22 (22) | 0.002 |

NOTE: Quality care denotes admissions satisfying study definition of quality care. Non-quality care denotes admissions not satisfying study definition of quality care.
