# Supplementary material for: Impact of Physician Specialty on Quality Care for Patients Hospitalized with Decompensated Cirrhosis
Source: PLoS One. 2015 Apr 2;10(4):e0123490. doi: 10.1371/journal.pone.0123490 (PMC4383455; doi:10.1371/journal.pone.0123490)
Supplement: S3 Table — (DOCX) [file pone.0123490.s003.docx]

Table S3. Provision of quality care in intensivist-managed admissions that did or did not receive gastroenterology (GI) consultation

|  | Quality care(%) | P-value |
| --- | --- | --- |
| **Overall** |  |  |
| With GI consultation | 55/77 (71.4) |  |
| Without GI consultation | 6/8 (75) | >0.99 |
|  |  |  |
| **Refractory Ascites** |  |  |
| With GI consultation | 1/3 (33.3) |  |
| Without GI consultation | 0/0 (0) | >0.99 |
|  |  |  |
| **Upper GI Bleeding** |  |  |
| With GI consultation | 49/68 (72.1) |  |
| Without GI consultation | 0/0 (0) | >0.99 |
|  |  |  |
| **Hepatic Encephalopathy** |  |  |
| With GI consultation | 4/4 (100) |  |
| Without GI consultation | 6/6( 100) | >0.99 |
|  |  |  |
| **Spontaneous Bacterial Peritonitis** |  |  |
| With GI consultation | 1/2( 50) |  |
| Without GI consultation | 0/2 (0) | >0.99 |
|  |  |  |

NOTE: Quality care denotes admissions satisfying study definition of quality care.
