# Supplementary material for: Impact of Physician Specialty on Quality Care for Patients Hospitalized with Decompensated Cirrhosis
Source: PLoS One. 2015 Apr 2;10(4):e0123490. doi: 10.1371/journal.pone.0123490 (PMC4383455; doi:10.1371/journal.pone.0123490)
Supplement: S4 Table — (DOCX) [file pone.0123490.s004.docx]

Table S4. Provision of quality care in hospitalist-managed admissions that did or did not receive gastroenterology (GI) consultation

|  | Quality care(%) | P-value |
| --- | --- | --- |
| **Overall** |  |  |
| With GI consultation | 46/78 (60) |  |
| Without GI consultation | 40/74 (54) | 0.62 |
|  |  |  |
| **Refractory Ascites** |  |  |
| With GI consultation | 6/12 (50) |  |
| Without GI consultation | 13/24 (54.2) | >0.99 |
|  |  |  |
| **Upper GI Bleeding** |  |  |
| With GI consultation | 11/24 (45.8) |  |
| Without GI consultation | 0/0(0) | >0.99 |
|  |  |  |
| **Hepatic Encephalopathy** |  |  |
| With GI consultation | 20/23 (86.9) |  |
| Without GI consultation | 26/50 (52) | 0.004 |
|  |  |  |
| **Spontaneous Bacterial Peritonitis** |  |  |
| With GI consultation | 9/19 (47.4) |  |
| Without GI consultation | 1/10 (10) | 0.10 |

NOTE: Quality care denotes admissions satisfying study definition of quality care.
