# Supplementary material for: Impact of Physician Specialty on Quality Care for Patients Hospitalized with Decompensated Cirrhosis
Source: PLoS One. 2015 Apr 2;10(4):e0123490. doi: 10.1371/journal.pone.0123490 (PMC4383455; doi:10.1371/journal.pone.0123490)
Supplement: S5 Table — (DOCX) [file pone.0123490.s005.docx]

Table S5. Proportion of adverse clinical outcomes in relation to adherence to specific quality measures

|  | Yes (%) | No (%) | P-value |
| --- | --- | --- | --- |
| **Refractory Ascites** |  |  |  |
| *Timely diagnostic paracentesis* |  |  |  |
| In-hospital complications | 2/23 (8.7) | 7/15 (46.6) | 0.02 |
| ICU transfer, where eligible | 2/21 (9.5) | 2/15 (13.3) | >0.99 |
| Length of hospital stay, median | 3 | 5 | 0.53 |
| In-hospital death | 1/23 (4.34) | 4/15 (25) | 0.14 |
|  |  |  |  |
| *Cell count, differential and culture on ascitic fluid* |  |  |  |
| In-hospital complications | 6/30 (20) | 1/2 (50) | 0.40 |
| ICU transfer, where eligible | 3/26 (11.5) | 1/2 (50) | 0.44 |
| Length of hospital stay, median | 3 | - | >0.99 |
| In-hospital death | 3/30 (10) | 1/2 (50) | 0.24 |
|  |  |  |  |
| *Management with diuretics and Na-restriction if renal function normal* |  |  |  |
| In-hospital complications | 2/25 (8) | 1/4 (25) | 0.37 |
| ICU transfer, where eligible | 1/24 (4.16) | 1/3 (33.3) | 0.21 |
| Length of hospital stay, median | 2 | 2.5 | 0.91 |
| In-hospital death | 1/25 (4) | 1/4 (25) | 0.26 |
|  |  |  |  |
| **Upper GI Bleeding** |  |  |  |
| *EGD within 12 hours** |  |  |  |
| In-hospital complications | 15/68 (22) | 4/24 (16.6) | 0.77 |
| ICU transfer, where eligible | 5/14 (35.7) | 1/10 (10) | 0.34 |
| Length of hospital stay, median | 4 | 3 | 0.68 |
| In-hospital death | 2/68 (2.94) | 0/24 (0) | >0.99 |
|  |  |  |  |
| *Antibiotics* |  |  |  |
| In-hospital complications | 17/77 (22.1) | 2/15 (13.3) | 0.73 |
| ICU transfer, where eligible | 5/15 (33.3) | 1/9 (11.1) | 0.35 |
| Length of hospital stay, median | 4 | 3 | 0.04 |
| In-hospital death | 1/77 (1.29) | 1/15 (6.66) | 0.30 |
|  |  |  |  |
| *EGD or TIPS if re-bleeding* |  |  |  |
| In-hospital complications | 5/5 (100) | 0/0 (0) | - |
| ICU transfer, where eligible | 1/1 (100) | 0/0 (0) | - |
| Length of hospital stay, median | 10 | - | - |
| In-hospital death | 1/5 (20) | 0/0 (0) | - |
|  | Yes (%) | No (%) | P-value |
| *Octreotide if variceal bleeding* |  |  |  |
| In-hospital complications | 16/58 (27.6) | 1/5 (20) | >0.99 |
| ICU transfer, where eligible | 1/5 (20) | 1/3 (33.3) | >0.99 |
| Length of hospital stay, median | 5 | 5 | 0.72 |
| In-hospital death | 1/58 (1.72) | 0/5 (0) | >0.99 |
|  |  |  |  |
| *Endoscopic band ligation if variceal bleeding* |  |  |  |
| In-hospital complications | 9/43 (20.9) | 0/0 (0%) | - |
| ICU transfer, where eligible | 0/5 (0) | 0/0 (0%) | - |
| Length of hospital stay, median | 4.5 | - | - |
| In-hospital death | 0/43 (0) | 0/0 (0%) | - |
|  |  |  |  |
| **Hepatic Encephalopathy** |  |  |  |
| *Search for reversible factors* |  |  |  |
| In-hospital complications | 8/55 (14.5) | 5/28 (17.9) | 0.75 |
| ICU transfer, where eligible | 4/46 (8.69) | 4/28 (14.3) | 0.47 |
| Length of hospital stay, median | 4 | 4 | 0.57 |
| In-hospital death | 1/55 (1.82) | 0/28 (0) | >0.99 |
|  |  |  |  |
| *Paracentesis if ascites documented* |  |  |  |
| In-hospital complications | 3/14 (21.4) | 1/4 (25) | >0.99 |
| ICU transfer, where eligible | 2/11 (18.2) | 0/4 (0) | >0.99 |
| Length of hospital stay, median | 5 | 4 | 0.37 |
| In-hospital death | 1/14 (7.14) | 0/4 (0) | >0.99 |
|  |  |  |  |
| *Treatment with lactulose and/or rifaximin if persistent* |  |  |  |
| In-hospital complications | 2/24 (8.33) | 1/2 (50) | 0.22 |
| ICU transfer, where eligible | 2/23 (8.69) | 0/2 (0) | >0.99 |
| Length of hospital stay, median | - | - | - |
| In-hospital death | 0/24 (0) | 0/2 (0) | >0.99 |
|  |  |  |  |
| **Spontaneous Bacterial Peritonitis** |  |  |  |
| *Diagnostic paracentesis* |  |  |  |
| In-hospital complications | 7/28 (25) | 0/5 (0) | 0.56 |
| ICU transfer, where eligible | 0/23 (0) | 1/5 (20) | 0.18 |
| Length of hospital stay, median | 5 | 13 | 0.02 |
| In-hospital death | 2/28 (7.14) | 1/5 (20) | 0.40 |
|  |  |  |  |
| *Antibiotics within 6 hours* |  |  |  |
| In-hospital complications | 6/30 (20) | 1/3 (33.3) | 0.52 |
|  | Yes (%) | No (%) | P-value |
| ICU transfer, where eligible | 1/25 (4) | 0/3 (0) | >0.99 |
| Length of hospital stay, median | 5 | 10 | 0.50 |
| In-hospital death | 2/30 (6.66) | 1/3 (33.3) | 0.26 |
|  |  |  |  |
| *Albumin within 6 hours, when indicated* |  |  |  |
| In-hospital complications | 1/7 (14.2) | 5/18 (27.7) | 0.63 |
| ICU transfer, where eligible | 0/6 (0) | 1/14 (7.1) | >0.99 |
| Length of hospital stay, median | 5 | 6 | 0.67 |
| In-hospital death | 0/7 (0) | 3/18 (16.7) | .53 |

NOTE: Yes represents admissions adhering to a specific quality measure. No represents admissions not adhering to a specific quality measure. Abbreviations: EGD, endoscopy; TIPS, transjugular portosystemic shunt.

*In one case, EGD was not performed because of patient refusal. There were no associated in-hospital complications associated with this case, including death, and the length of hospitalization was 7 days.
